# Comparison of the Pollen Deposition and Carrying Efficiency of Four Wild Pollinators for Oil-Seed Camellia Trees

**DOI:** 10.3390/insects17020153

**Published:** 2026-01-30

**Authors:** Zijian Li, Yu Qiao, Mvchir Huyun, Yan Li, Wei Zhang, Yue Ying, Jinping Shu

**Affiliations:** 1 Research Institute of Subtropical Forestry, Chinese Academy of Forestry, Hangzhou 311400, China; lzj030520@163.com (Z.L.); zhoubai0913@gmail.com (Y.Q.); mvchirhy@163.com (M.H.); liyan941030@126.com (Y.L.); zwlzhi@126.com (W.Z.); zhczscxf@163.com (Y.Y.); 2 College of Forestry, Nanjing Forestry University, Nanjing 210037, China

**Keywords:** *Camellia oleifera*, pollinating insects, functional trait, body hair, pollen load

## Abstract

To investigate how insect hair morphology influences pollination effectiveness, this study examined four common wild pollinators in *Camellia oleifera* plantations: two bee species (*Colletes gigas* and *Apis cerana*) and two hornet species (*Vespa velutina* and *Vespa soror*). We systematically measured hair length, hair density, and pollen loads on four body regions (head, thorax, abdomen, and legs). The results indicated that the following: (1) *C. gigas* possessed significantly longer and denser hairs across all body parts, especially on the legs, compared to the other three species. (2) Both the pollen load per body part and the total pollen load were markedly higher in *C. gigas* than in the other pollinators. The two hornet species did not differ significantly from *A. cerana* in pollen load, and even exceeded it in certain traits such as head hair length. (3) Correlation analysis revealed a significant positive relationship between total pollen load and both hair length (ρ = 0.545, *p* < 0.01) and hair density (ρ = 0.391, *p* < 0.01). Pollen loads on different body regions were also strongly positively correlated, suggesting functional synergy across the insect’s surface. Leg pollen load correlated positively with head and leg hair length, but negatively with head hair density. Notably, leg hair length and density showed a unique positive correlation, highlighting region-specific morphological adaptation. (4) Cluster analysis separated *C. gigas* from the other three species, which grouped together. In conclusion, hair length and density—particularly on the legs—are key morphological traits underpinning pollen-carrying efficiency in these pollinators. *C. gigas* demonstrates superior hair morphology and pollen-carrying performance, supporting its role as an effective pollinator of *C. oleifera*. This study provides a trait-based framework for identifying dominant pollinators and underscores that evaluating species with complex ecological roles, such as hornets, requires integrating morphological traits with broader behavioral and community contexts.

## Simple Summary

*Camellia oleifera* is a vital woody oil crop in China, with its pollination process heavily dependent on insect vectors. Notably, during its flowering period—which coincides with low-temperature conditions—the crop exhibits a heightened reliance on pollinators, whose activity levels and pollination efficiency directly dictate the final yield of *C. oleifera*. This study explored the relationship/correlations of body hair traits (length and density) of four commonly found pollinators of *C. oleifera*, *Colletes gigas*, *Apis cerana*, *Vespa velutina*, and *Vespa soror*—with their pollen-carrying capacity. The results showed that *C. gigas* had significantly longer and denser leg hair, carrying far more pollen than other species. A clear positive correlation was found between the overall pollen-carrying capacity of insects and the length/density of their hair. The study confirmed that the high density and length of leg hair are key functional traits for pollinators to carry pollen efficiently, making *C. gigas* the most suitable pollinator for *C. oleifera*. This achievement provides a scientific basis for screening dominant pollinators of *C. oleifera*, which helps improve pollination efficiency and yield, and is of great significance for ensuring the supply of high-quality edible oil.

## 1. Introduction

Pollinators play a critical role in ecosystems by facilitating pollination processes that ensure the reproduction and survival of hundreds of thousands of plant species worldwide, thereby profoundly influencing global food security and ecological stability. According to the latest global assessment, approximately 80% to 85% of angiosperms rely on invertebrate or vertebrate pollinators, with insects constituting the dominant group among animal pollinators [1], and pollinating insects play pivotal role in maintaining biodiversity and ecosystem functionality. Functional traits are measurable morphological, physiological, or phenological characteristics at the organismal level that represent adaptive responses to environmental variations and host interactions, while simultaneously indicating an individual’s influence on ecosystem functioning [2]. Recent research has increasingly centered on the diversity of functional traits—particularly in plants—and their relationships with species diversity, environmental change, and community composition, alongside their critical ecosystem roles [3]. In contrast, key characteristics mediating multitrophic functional relationships in animals remain poorly understood, especially among terrestrial invertebrates [4]. As essential components of animal pollinators, pollinating insects have developed specialized morphological adaptations involved in the co-evolution with plants [5]. The hairs covering insect bodies serve as critical functional traits influencing thermoregulation, pollen collection, transfer efficiency, and pollination performance [3,6,7]. The hair covering hymenopteran integument function as critical morphological adaptations for pollen collection and pollination enhancement. Key hair traits—including morphology, length, and density—demonstrate strong functional linkages with pollen transfer efficiency across some angiosperm–pollinator systems [8,9]. Cullen et al. conducted quantitative analyses of pollen load and hair characteristics across 251 bee (Apoidea) and 95 fly (Diptera) pollinator specimens collected from McLaughlin Natural Reserve, California. Their research demonstrated that pollinators with densely setose integuments carried up to 35 times greater pollen loads than those with glabrous or sparsely setose bodies [10]. Goulnik et al. found that facial pollen load was positively correlated with both facial hair area and facial hair amount, and emphasized that the hair amount and area of pollinators are notable characteristics of pollination effectiveness [11]. Phillips et al. found that among 14 families of pollinators interacting with oilseed rape (*Brassica napus* L., 1753), the head hairs of pollinators are the primary body part for pollen transport [9]. Stavert et al. found that the amount of facial hair was the best predictor of total pollen load for 10 different pollinator species (either bees or flies) when interacting with Chinese cabbage [12]. Studies have found that the sex, taxonomic class, body size, and hair density of pollinating insects have a significant impact on the pollen load they carry [11,13,14,15,16,17]. Changes in pollinator traits also drive the differentiation of the taxonomic and phylogenetic composition of pollen loads. For instance, sex has been shown to affect the flower visitation preferences of bees [16]. Exploring the relationship between body hair and insect pollination efficiency is particularly important for evaluating the role of pollinating insects in ecosystems.

*Camellia oleifera* Abel, 1818, a primary woody oil crop in China, ranks among the world’s four major oil-bearing tree species alongside *Elaeis guineensis* Jacq., 1763, n*Olea europaea* L., 1753, and *Cocos nucifera* L., 1753 [18]. Tea oil derived from *C. oleifera* exhibits golden-to-pale-yellow coloration, exceptional purity, and crystal clarity. Recognized as the “Oriental *O. europaea* Oil,” it constitutes a premium-grade edible oil [19]. *C. oleifera* is indigenous to southern China and Southeast Asia, including Vietnam, Laos, Myanmar, and Assam State, India [20]. In China, wild populations occur in Hunan Province, Jiangxi Province, Hainan Province, and the Guangxi Zhuang Autonomous Region, with extensive cultivation throughout the Yangtze River Basin and southern regions [21]. This species exhibits tight mutualism with pollinators. As an obligate outcrossing tree, *C. oleifera* pollination depends critically on insects, exhibiting <3% fruit set through autogamy or anemophily [22,23]. Field surveys identify >90 potential pollinator species across four orders: Hymenoptera (71.2%), Coleoptera (14.1%), Diptera (9.3%), and Lepidoptera (5.4%), with bees constituting both the dominant (82% of visits) and most efficient pollen vectors, with bees quantitatively confirmed as the most efficient pollen vectors [24,25,26,27]. Hongying Li et al. found in their study that, from a spatial perspective, the abundance of wild bees was positively correlated with fruit set; however, fruit set was negatively correlated with the distance to the aggregation of large nearby hives [28]. Studies have shown that more than 40 species of insects can pollinate *C. oleifera* (oil-tea camellia), and *Colletes gigas* Cockerell, 1918, as a dominant pollinator, is frequently mentioned in various studies [29,30]. It is noteworthy that although Hymenopteran insects, primarily represented by *C. gigas*, have been confirmed as the most effective pollinators of *C. oleifera*, the relative contributions and adaptive mechanisms of other pollinator groups (such as wasps) in specific environments still require more in-depth quantitative research.

For common pollinating insects such as bees, pubescence plays a crucial role in plant pollination; however, as wasps also serve as important pollinators of *C. oleifera*, the morphological and functional differences in their pubescent structures require further in-depth investigation. This study selected four common pollinator species in *C. oleifera* plantations—*C. gigas*, *Vespa velutina* Lepeletier, 1836, *Apis cerana* Fabricius, 1793, and *Vespa soror* du Buysson, 1905—as research subjects. We conducted comparative analyses of hair length, density, and corresponding pollen load across distinct body regions (head, thoracic, abdominal, and legs) to elucidate trait-specific relationships. This investigation aims to decipher how hair characteristics govern pollinator pollen-carrying capacity and establish empirical criteria for screening superior *C. oleifera* pollinators. To investigate the relationship between different body parts of pollinators of *C. oleifera* (oil-tea camellia) and their pollen-carrying capacity, as well as the impact of hair traits on pollen-carrying capacity, this study proposes and addresses the following research questions: (a) What are the differences in pollen-carrying capacity among different body parts of the four pollinator species? (b) Are there differences in hair length and density between different body parts of the pollinators? (c) What is the impact of hair length and density on pollen-carrying capacity? We hypothesize that: pollinators carry far more pollen on their hind legs than other body parts; hair length and density differ significantly across body parts; and these hair traits are positively correlated with pollen load. This study aims to demonstrate the differences in pollen load capacity among pollinators and clarify that the hind legs of pollinators, as the primary pollen-carrying part, affect the pollen quantity carried by other parts, thereby influencing the overall pollination capacity of the insects.

## 2. Materials and Methods

### 2.1. Pollinator Collection and Preservation

Pollinator sampling was conducted at the Yong’an Mountain *C. oleifera* Abel plantation (119°55′ E, 29°52′ N) in Fuyang District, Hangzhou, Zhejiang Province. The site features a 10-year-old monoculture of *C. oleifera* var. oleifera two hectares in size. During peak pollinator activity hours (13:00–16:00 CST) on days with ambient temperatures ≥ 12 °C and clear skies, actively foraging hymenopterans were collected directly from *C. oleifera* blossoms. Small, non-defensive species (e.g., *A. cerana*) were captured using inverted 50 mL centrifuge tube confinement. Larger, aggressive taxa (*V. velutina*, *V. soror*) were collected via aerial netting. All specimens underwent immediate cryoanesthesia through liquid nitrogen immersion (−196 °C) for morphological preservation [28,30].

### 2.2. Species Identification Methods

Morphological characterization of the collected pollinator specimens was performed using a high-resolution, deep-focus 3D digital microscope (Keyence VHX-5000, Keyence, Osaka, Japan), with comprehensive photomicrographic documentation acquired during the observation process. Species-level identification was achieved through rigorous comparative morphological analysis against authoritative taxonomic descriptions contained within the following seminal works: Fauna Sinica (Insecta Vol. 20: Hymenoptera, Melittidae, Apidae); Economic Insect Fauna of China (Fasc. 9: Hymenoptera, Apoidea; Fasc. 30: Hymenoptera, Vespoidea); Pictorial Handbook of Forest Insects in Hunan (Ichneumonidae, Vespoidea, Apoidea); and Hymenoptera of Zhejiang (Vespoidea: Vespidae; Apoidea: Colletidae, Andrenidae, Halictidae, Megachilidae, Apidae). This systematic examination facilitated the precise selection of target pollinators. Subsequently, voucher specimens representing these taxonomically screened species were subjected to expert validation through submission to recognized taxonomic authorities for critical review and definitive confirmation [31].

### 2.3. Determination of Pollen Grain Numbers on Different Body Parts of Pollinators

Under a dissecting microscope, pollinator insects were dissected into four parts (head, thorax, abdomen, and legs) using forceps and a scalpel. Each part was placed into a separate 5 mL sample tube containing absolute ethanol for pollen elution. Pollen load was quantified using a hemocytometer. Prior to counting, sample tubes were vortexed for 20 s to create a homogeneous suspension. A micropipette was used to transfer 10 μL of the solution onto the hemocytometer, and a coverslip was applied. Pollen grains within the sample were counted under a microscope. The effective pollen load for each insect body part was calculated using the formula: Effective pollen load per insect part = Pollen count in 10 μL × 500. Four insect individuals were analyzed per species, with five repeated measurements taken for each body part per individual [32].

### 2.4. Measurement of Hair Length on Different Body Parts of Pollinators

Under a dissecting microscope, pollinator insects were dissected into four parts (head, thorax, abdomen, and legs) using forceps and a scalpel. Using the integrated measurement tools of the microscopic imaging system, five 0.1 mm^2^ areas were randomly selected on each body part for analysis. The length of 5–7 hair in each region was measured, and the average length of these measured hair was recorded as the representative hair length for that body part of the insect. To achieve more precise hair length measurements, small tissue sections were excised from the dissected body parts of the pollinators using a microknife. These sections were placed onto glass slides. Hair from the desired regions were gently scraped free using a microknife, separated using dissecting needles, and their lengths were measured using the measurement software of a digital microscope (providing extended depth of field). Given that the legs typically serve as the primary pollination organs, morphological photographs of the leg hairs were taken for each pollinator under a microscope. Four replicates were performed per insect species.

### 2.5. Measurement of Hair Density on Different Body Parts of Pollinators

Following the methodology of Roquer-Beni et al., we randomly selected five approximately 0.1 mm^2^ areas on each of the aforementioned four body parts and counted the number of hairs within each area [3]. In certain cases, particularly for species with high hair density and specimens where hair became clumped due to handling during capture or preservation, it was easier to count hair at their insertion points—which typically appear as micropores on the cuticle. Moreover, counting these micropores has an additional advantage: it works for specimens that have shed hair, allowing measurement of the original hair coverage. The results of these five measurements were used to calculate the average hair density for each body part. In some species, the distribution of hair was clearly heterogeneous across a given body part. In such instances, regions of approximately 0.1 mm^2^ (square millimeters) dominated by hair from distinct body parts were sampled separately. Subsequently, the overall average hair density was computed via a weighted average approach, with calculations based on the proportional area occupied by hair from each body part. Four replicates were performed for each insect species to ensure data reliability [31].

### 2.6. Data Statistics and Analysis

Data analysis was performed using SPSS 22.0. For comparisons among pollinator species (interspecific comparisons), one-way analysis of variance (ANOVA) was conducted using the mean value of each measured trait (hair length, hair density, pollen load) per individual insect as the independent data point (*n* = 4 individuals per species). For comparisons among different body parts within the same species (intraspecific comparisons), a repeated-measures ANOVA was employed, with ‘individual’ treated as the random effect to account for non-independence of measurements from the same insect. Pearson correlation analysis was performed on data averaged per individual per body part to explore the relationships between insect hair morphology and pollen-carrying capacity, specifically analyzing the correlations between hair length and density for each body part and the corresponding pollen load. Based on comprehensive parameters of hair number, density, and length, principal component analysis (PCA) was conducted, further revealing the differentiation patterns in morphological traits related to pollen carriage among the four pollinator species from a multivariate perspective. All graphs and charts were created and visualized using R (Version 4.4.1) and Origin 2024.

## 3. Results

### 3.1. Species Identification

**Figure 1:**
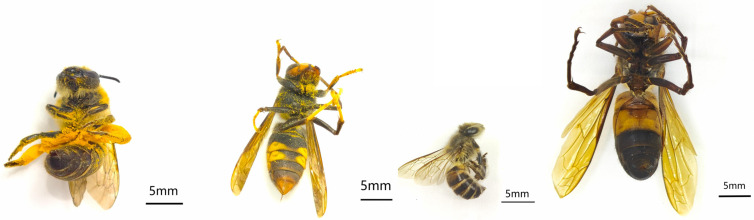
Pictures of four pollinating insects. From left to right, they are *Colletes gigas*, *Vespa velutina*, *Apis cerana*, and *Vespa soror.* Note: This figure is intended to illustrate the morphology of the insects and the general overview of pollen deposition on their body surface. The photographs are not scaled to a uniform proportion. For accurate morphological comparisons, please refer to the subsequent measurement data figures and charts.

Morphological identification of the pollinator insects collected from the *C. oleifera* plantation at Yong’an Mountain, Fuyang District, Hangzhou City, Zhejiang Province, revealed that the majority belonged to *A. cerana* and *C. gigas*. The collection also included a smaller number of vespid wasps, ants, and Germ Fly. Subsequently, four species of pollinators were selected as the experimental subjects for this study: *C. gigas*, *V. velutina*, *A. cerana* and *V. soror* (Figure 1).

### 3.2. Comparison of Hair Length Differences Among Pollinator Species

**Figure 2:**
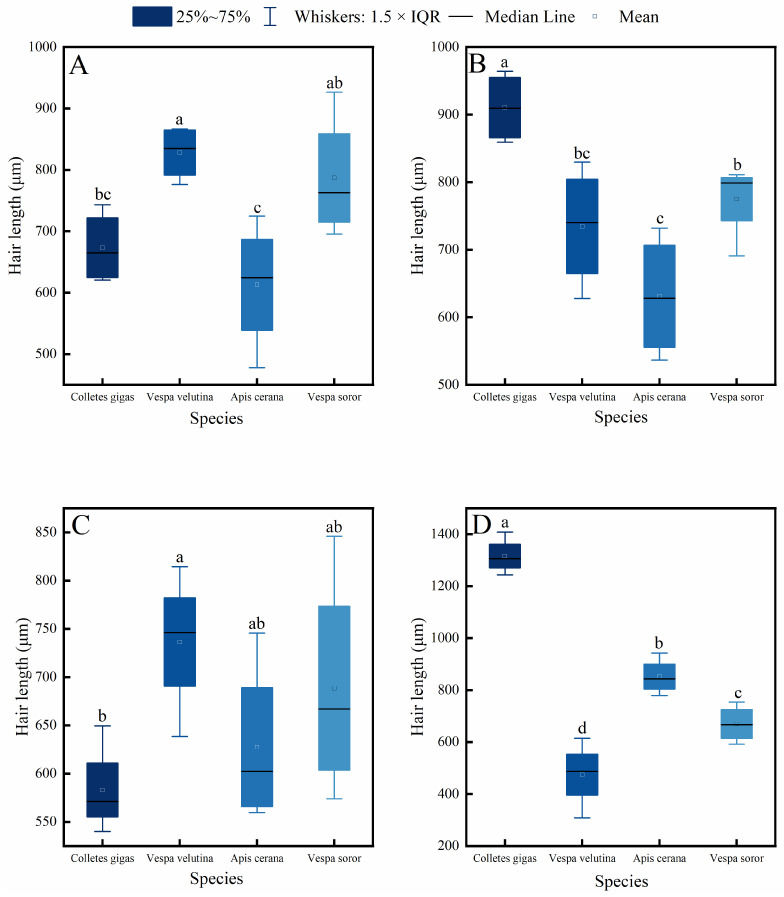
Comparison of hair length among four pollinator species across different body parts. (**A**) Head, (**B**) Thorax, (**C**) Abdomen, (**D**) Leg. Note: Different lowercase letters indicate statistically significant differences among species within the same body part (*p* < 0.05). *X*-axis: species (*C. gigas*, *V. velutina*, *A. cerana*, *V. soror*). *Y*-axis: hair length (μm).

**Figure 3:**
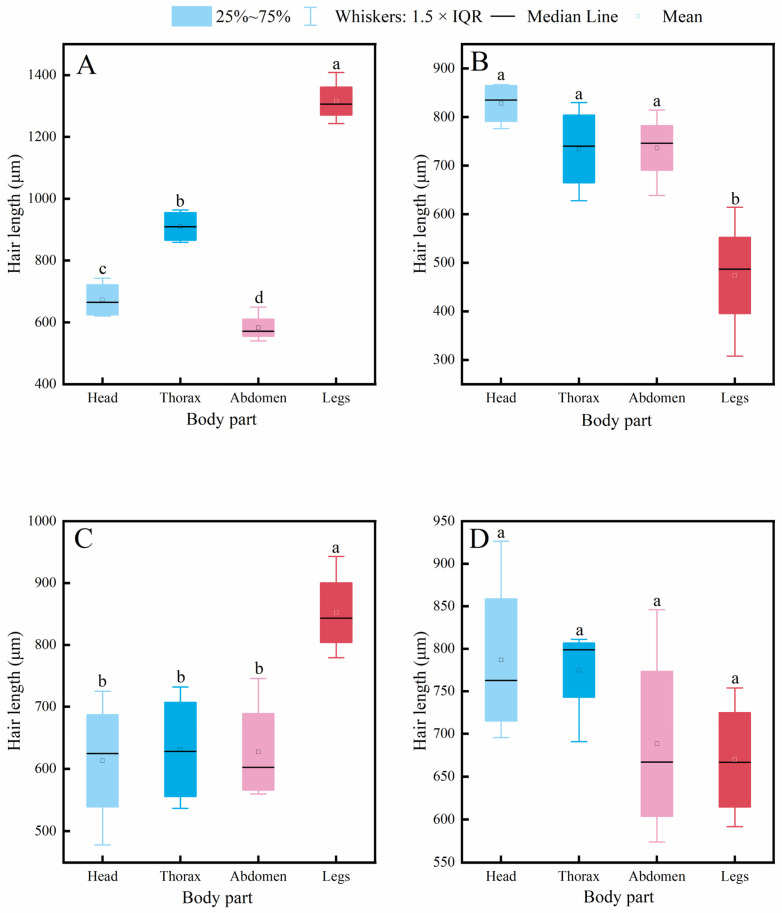
Intraspecific variation in hair length across different body parts of four pollinator species. (**A**) *C*. *gigas*, (**B**) *V*. *velutina*, (**C**) *A*. *cerana*, (**D**) *V*. *soror.* Note: Different lowercase letters indicate statistically significant differences among body parts within the same species (*p* < 0.05). *X*-axis: body part (Head, Thorax, Abdomen, Leg). *Y*-axis: hair length (μm).

Measurements and comparisons of hair length across four key body parts (head, thorax, abdomen, and leg) in the four pollinator species—*C. gigas*, *V. velutina*, *A. cerana*, and *V. soror*—revealed significant interspecific differences and distinct intraspecific variation patterns (Figure 2 and Figure 3).

Interspecific comparisons (Figure 2) showed significant differences in hair length for the same body part among the species. *C. gigas* exhibited the longest hair across all measured body parts, with hair length on its legs significantly exceeding that of the other three species. The head hair length of both hornet species (*V. velutina* and *V. soror*) was significantly greater than that of *A. cerana* and showed no significant difference from *C. gigas*. In contrast, *A. cerana* generally had the shortest hair among the four species, particularly on the thorax and legs.

Intraspecific comparisons (Figure 3) revealed different patterns of variation among body parts within each species. A common feature observed in *C. gigas*, *V. velutina*, and *A. cerana* was that hair length on the legs was significantly greater than on the head, consistent with the functional role of legs as primary structures for pollen handling. Specifically, *C. gigas* displayed a clear gradient: leg > thorax > abdomen ≈ head. *V. velutina* showed a pattern where hair length on the legs and thorax was similar and both were longer than on the abdomen and head. For *A. cerana*, a significant difference was found only between the legs and the head, with other body parts having comparable hair lengths. Notably, *V. soror* was the only species in which no significant differences in hair length were detected among its four body parts, indicating a relatively uniform distribution of hair.

### 3.3. Comparison of Hair Density Differences Among Pollinator Species

**Figure 4:**
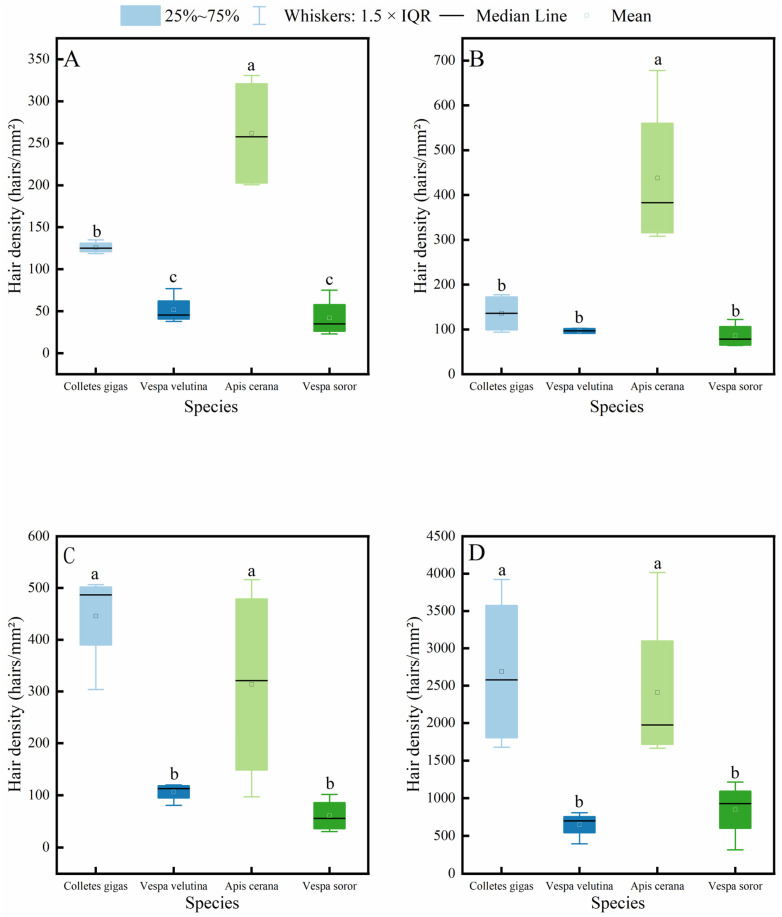
Comparison of hair density on different body parts among four pollinator species. (**A**) Head, (**B**) Thorax, (**C**) Abdomen, (**D**) Leg. Note: Different lowercase letters indicate statistically significant differences among species within the same body part (*p* < 0.05). *X*-axis: species (*C. gigas*, *V. velutina*, *A. cerana*, *V. soror*). *Y*-axis: hair density (hairs/mm^2^).

It is generally accepted that the density of body hair in insects is closely related to their pollen-carrying capacity, and thus, it is often used as an important indicator for evaluating the pollination efficiency of pollinator insects. The results of this study (Figure 4) show that the hair density on the head, thorax, abdomen, and legs of *A. cerana* was significantly higher than that of *V. velutina* and *V. soror*. Except for the thoracic hair density, which showed no significant difference from that of the two hornet species, the hair density in *C. gigas* was also significantly higher than that of the hornets in all other body parts. Significant differences between *C. gigas* and *A. cerana* were only observed in the hair density on the head and thorax, and *A. cerana* exhibited the highest hair density across all body parts among the four insects. In addition, no statistically significant differences were found between *V. velutina* and *V. soror* in any corresponding body part.

**Figure 5:**
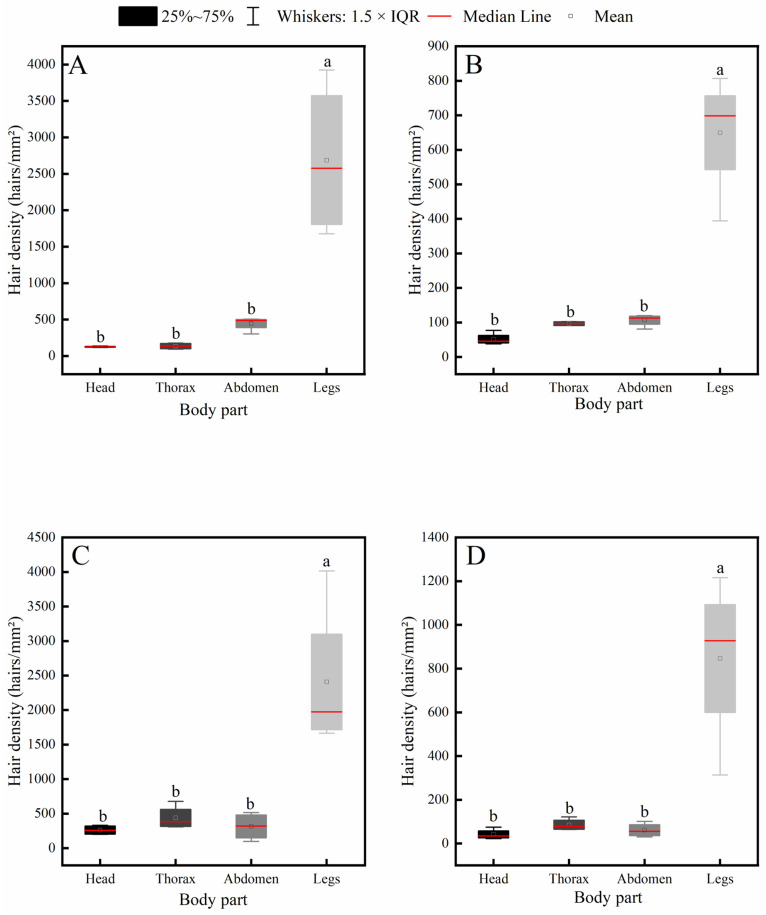
Intraspecific variation in hair density across different body parts of four pollinator species. (**A**) *C*. *gigas*, (**B**) *V*. *velutina*, (**C**) *A*. *cerana*, (**D**) *V*. *soror.* Note: Different lowercase letters indicate statistically significant differences among body parts within the same species (*p* < 0.05). *X*-axis: body part (Head, Thorax, Abdomen, Leg). *Y*-axis: hair density (hairs/mm^2^).

Further intraspecific comparison results (Figure 5) indicated that all four insect species exhibited a consistent distribution pattern of body hair: hair density on the legs was significantly higher than that on the head, thorax, and abdomen, while no significant differences were observed among the head, thorax, and abdomen.

### 3.4. Pollen Load Comparisons Among Pollinator Species

**Figure 6:**
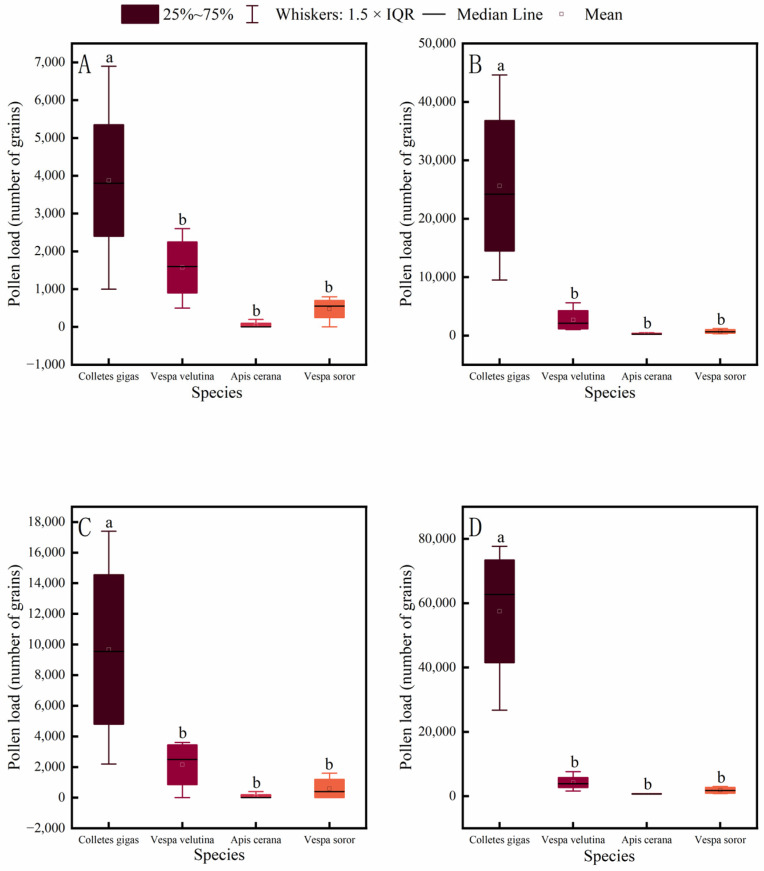
Comparison of pollen load on different body parts among four pollinator species. (**A**) Head, (**B**) Thorax, (**C**) Abdomen, (**D**) Leg. Note: Different lowercase letters indicate statistically significant differences among species within the same body part (*p* < 0.05). *X*-axis: species (*C. gigas*, *V. velutina*, *A. cerana*, *V. soror*). *Y*-axis: pollen load (number of grains).

Pollen-carrying capacity of pollinator insects is a key indicator for evaluating their pollination efficiency. This study measured the pollen loads on different body parts of four common pollinator species in an oil-tea plantation. The results showed that the pollen load on each body part of *C. gigas* was significantly higher than that of *A. cerana*, *V. soror*, and *V. velutina* (Figure 6). In contrast, no significant differences were found in pollen loads among *V. velutina*, *A. cerana*, and *V. soror* for any corresponding body part, with *A. cerana* carrying the lowest pollen loads overall.

**Figure 7:**
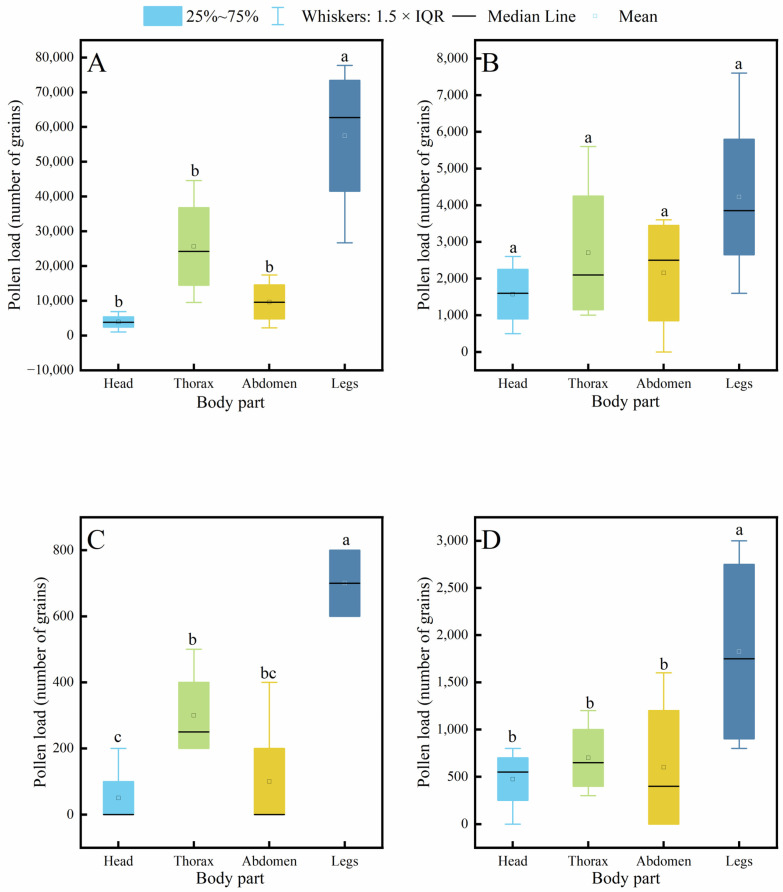
Intraspecific variation in pollen load across different body parts of four pollinator species. (**A**) *C*. *gigas*, (**B**) *V*. *velutina*, (**C**) *A*. *cerana*, (**D**) *V*. *soror.* Note: Different lowercase letters indicate statistically significant differences among body parts within the same species (*p* < 0.05). *X*-axis: body part (Head, Thorax, Abdomen, Leg). *Y*-axis: pollen load (number of grains).

Further analysis of pollen distribution across body parts within each species revealed (Figure 7) that in *C. gigas*, the legs carried a significantly higher pollen load than the head, thorax, or abdomen, while no significant differences were observed among the latter three parts, with the head carrying the least pollen. In contrast, *V. velutina* showed no significant differences in pollen loads among different body parts. For A. cerana, the legs carried significantly more pollen than other parts, and the thorax also carried significantly more pollen than the head. Similarly, in *V. soror*, the pollen load on the legs was significantly higher than that on all other body parts.

**Figure 8:**
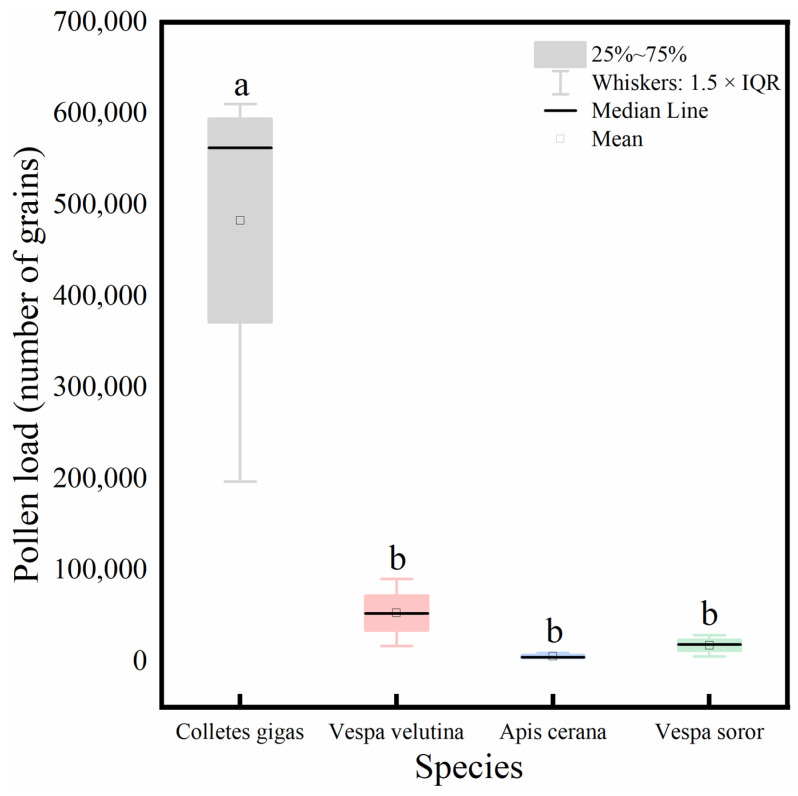
Comparison of total pollen load carried by four pollinator species. Note: Different lowercase letters above the bars indicate statistically significant differences among species at *p* < 0.05. *X*-axis: species (*C. gigas*, *V. velutina*, *A. cerana*, *V. soror*). *Y*-axis: pollen load (number of grains).

Analysis of the total pollen load carried by the four pollinator species (Figure 8) showed that *C. gigas* carried a significantly higher total pollen quantity than the other three species, among which no significant differences were observed.

### 3.5. Relationship Between Pollinator Hair Length, Density, and Pollen Load

**Figure 9:**
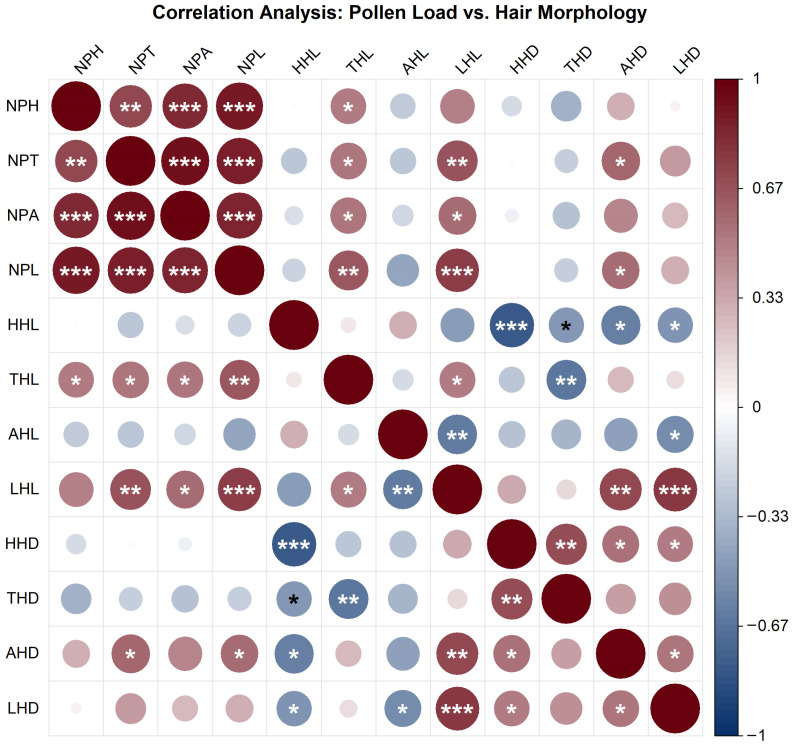
Correlation heatmap between pollen loads and hair morphological traits in pollinator insects. Note: The color gradient represents Pearson correlation coefficients (ranging from −1 to 1; dark red indicates strong positive correlation, dark blue indicates strong negative correlation). Circle size is proportional to the absolute value of the correlation coefficient. Statistical significance is denoted as follows: * *p* < 0.05, ** *p* < 0.01, *** *p* < 0.001. The figure illustrates the strength and significance of linear associations between pollen loads and hair length/density across different body regions.

The correlation between pollen loads on different body parts of pollinator insects and hair morphological traits (length and density) was investigated in this study (Figure 9). The results showed strong positive correlations among pollen loads from different body parts. Specifically, the correlations between head pollen load (NPH), thorax pollen load (NPT), abdomen pollen load (NPA), and leg pollen load (NPL) were all highly significant (*p* < 0.001), indicating a pronounced synergistic effect in pollen-carrying capacity across different body regions of the insects.

Multiple significant positive correlations were observed between pollen loads and hair length. In particular, leg pollen load (NPL) was significantly positively correlated with both head hair length (HHL) and leg hair length (LHL) (*p* < 0.001). Pollen loads on the head, thorax, and abdomen were also positively correlated with leg hair length (LHL) (*p* < 0.01 or *p* < 0.001). In contrast, the associations between pollen loads and hair density were predominantly negative. Only leg pollen load (NPL) showed a significant negative correlation with head hair density (HHD) (*p* < 0.001), while no widespread significant associations were found between other pollen load metrics and hair density.

Within the same body region, hair length and density were generally negatively correlated. For instance, significant negative correlations were found between head hair length (HHL) and head hair density (HHD) (*p* < 0.001), as well as between thorax hair length (THL) and thorax hair density (THD) (*p* < 0.01). A significant negative correlation was also observed between abdomen hair length (AHL) and abdomen hair density (AHD) (*p* < 0.05). Notably, leg hair length (LHL) and leg hair density (LHD) exhibited a significant positive correlation (*p* < 0.001), reflecting region-specific morphological characteristics.

In summary, these findings suggest a synergistic relationship in pollen-carrying function across different insect body regions, with a prevalent positive association with hair length—particularly leg hair length, which appears to be a key morphological determinant of pollen-carrying capacity. Conversely, pollen loads showed weak and mostly negative correlations with hair density, implying a possible trade-off between morphological structure and functional capacity within limited body surface area. The distinct correlation patterns observed in leg morphology and pollen-carrying function suggest that legs may possess specialized structural and functional adaptations in the pollination process. This study elucidates the complex relationships between body surface morphology and pollination function in pollinator insects, providing a foundation for further understanding of morphological adaptations and their ecological roles in pollination.

To further explore the relationship between insect body surface morphology and pollination function at an overall level, Pearson correlation analysis was performed on hair length, hair density, and pollen load across all insect subjects, with the significance level set at *p* < 0.05. The results (Table 1) showed that pollen load was significantly positively correlated with both hair length (ρ = 0.545) and hair density (ρ = 0.391) at the 0.01 level (two-tailed). Meanwhile, hair length and hair density also exhibited a significant positive correlation (ρ = 0.387) at the 0.01 level (two-tailed). These findings indicate that, at the overall level, both hair length and hair density are statistically significantly and positively associated with pollen-carrying capacity. Therefore, these two morphological traits may serve as important reference indicators for screening dominant pollinator insects in oil-tea plantations. Together with the previous part-specific analysis, these results reveal the multi-layered influence of body surface morphology on pollination function.

### 3.6. Species Differentiation Based on Pollen Load and Related Morphological Traits

**Figure 10:**
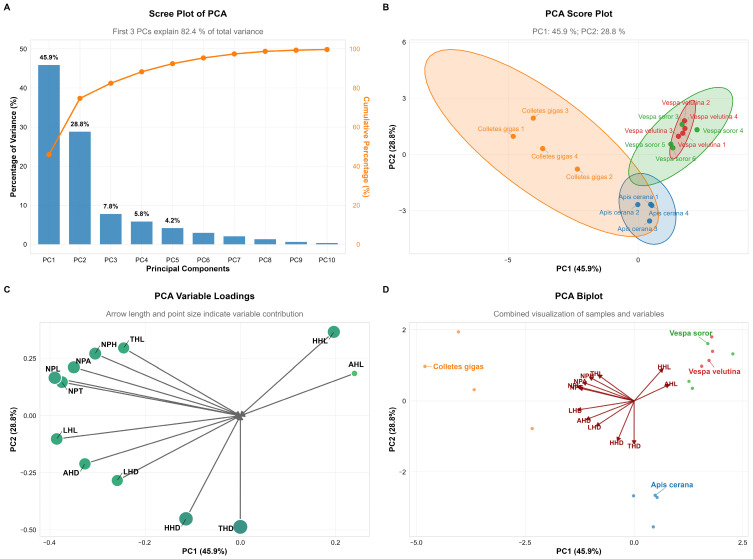
Principal Component Analysis (PCA) of pollen load, hair length, and hair density across body parts of pollinator insects. Note: (**A**) *Scree Plot*: Displays the variance explained by each principal component and the cumulative contribution rate. (**B**) *Score Plot*: Shows the sample clustering of four pollinator insect species based on PC1 and PC2, with colored ellipses representing 95% confidence intervals. (**C**) *Variable Loadings Plot*: The length of arrows and size of points reflect the contribution intensity of traits (e.g., pollen load, hair length/density) to PC1 and PC2. (**D**) *Biplot*: Integrates sample distribution and variable loadings to intuitively visualize the association between species clustering and traits.

Principal component analysis (PCA) revealed that the first two principal components (PC1 and PC2) together explained 74.7% of the total variance in pollen load and related morphological traits (PC1: 45.9%; PC2: 28.8%), indicating that these components effectively captured the differences in pollen-carrying capacity and associated morphological structures among the studied species (Figure 10A).

In terms of species distribution, Apis cerana and *C. gigas* showed relatively high scores on PC1 (−0.45 and −0.40, respectively), reflecting their well-developed body hair morphology, which aligns with their efficient pollen-collecting behavior. In contrast, hornet species (*V. velutina* and *V. soror*) generally exhibited lower scores on PC1 (ranging from −0.55 to −2.40), suggesting less developed body hair. This may be related to their non-obligatory pollination behavior and comparatively smoother body surface. Notably, the series of *V. soror* samples displayed a clear gradient in PC1 scores, indicating potential continuous intraspecific variation in the relevant morphological traits (Figure 10B).

Regarding morphological variables, AHL, NPH, and THL contributed strongly to PC1, with high negative loadings (−0.40, −0.30, and −0.20, respectively), highlighting their significant role in species differentiation. THD and HHD also showed moderate negative loadings on PC1. These results suggest that insect body hair structures, particularly hair length and density on the abdomen, thorax, and head, are closely associated with pollen adhesion and transport functionality (Figure 10C,D).

In summary, PCA revealed functional morphological differentiation among pollinator species in terms of pollen-carrying adaptation. Bee species exhibited body hair structures more suited for pollen attachment, whereas hornet species showed relatively weaker development in these traits.

### 3.7. Comparison of Leg Hair Morphology Among Pollinators

**Figure 11:**
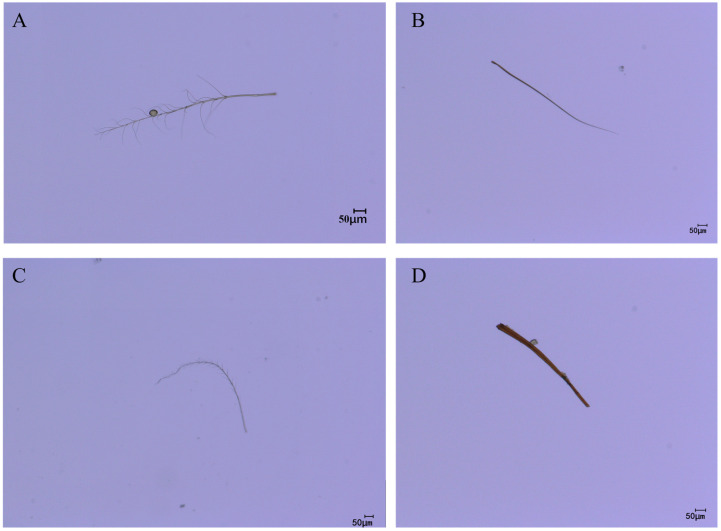
Leg hair morphology of different pollinators. Note: (**A**) leg hair morphology of *C. gigas*; (**B**) leg hair morphology of *V. velutina*; (**C**) leg hair morphology of *A. cerana*; (**D**) leg hair morphology of *V. soror*.

As the legs typically serve as the primary organs for pollen collection and transport during pollination, this study conducted microscopic observations of the leg hair morphology of the four pollinator species to complement the quantitative measurement data. Microscopic images revealed distinct interspecific differences in leg hair morphology among the pollinators (Figure 11). *C. gigas* possessed dense, elongated, and highly plumose (branched) setae on its legs, forming a complex structure with a large surface area highly conducive to pollen adherence (Figure 11A). In contrast, *V. velutina* exhibited relatively sparse, slender, and predominantly unbranched setae on its legs (Figure 11B). *A. cerana* displayed leg setae of moderate length and a simpler structure compared to the plumose hairs of *C. gigas*, with shorter branches (Figure 11C). The hair morphology of *V. soror* was similar to that of *V. velutina*, being linear and unbranched (Figure 11D). These direct morphological observations corroborate the quantitative differences in leg hair length, density, and associated pollen loads reported in Section 3.2, Section 3.3 and Section 3.4, providing a structural basis for explaining the variations in pollen-carrying efficiency among the different species.

## 4. Discussion

This study systematically investigated the relationship between hair characteristics and pollen load efficiency in four pollinators of *C. oleifera*. It was found that the hindleg hair length and density of *C. gigas* were significantly higher than those of other species, and its total pollen load was 8–12 times higher than that of *V. velutina*, *A. cerana*, etc. [31]. Pearson correlation analysis revealed that total pollen load was positively correlated with hair length (ρ = 0.545, *p* < 0.01) and density (ρ = 0.391, *p* < 0.01)—a finding consistent with Roquer-Beni, Stavert, et al., whose studies demonstrate that insect hair function as the primary sites for pollen attachment [3,12]. This result is consistent with Cullen et al., who found that pollinators with densely setose integuments carried up to 35 times greater pollen loads than those with glabrous or sparsely setose bodies [10], and also echoes the theory proposed by Stavert et al. that “hair density is a key factor for pollen attachment” [12].

From the perspective of functional morphology, the plumose branched hairs of *C. gigas* may form interlocks with the spinose exine of *C. oleifera* pollen through a mechanical chimeric mechanism. It is suggested that the scale matching between their branch spacing and pollen particle size could significantly enhance attachment efficiency [12]. To further elucidate the association between body hair morphology and pollen-carrying function within the specific context of *C. oleifera* pollination, this study investigated key functional traits in both pollinators and vespid wasps. It demonstrates that hair length and density are significant morphological predictors of pollen load across the studied pollinator assemblage [3,12]. It is worth noting that the hindleg hair of *C. gigas* include plumose branched long hair, long terminal-branched hair, and unbranched conical long hair, and this morphological diversity may enhance pollen capture by increasing surface area and mechanical friction [31]. The flowering period of *C. oleifera* (October to January of the following year) is often accompanied by a shortage of pollinators due to low temperatures [33,34]. The high hair density of *C. gigas* may serve a dual function of thermoregulation and pollination. Studies have shown that insect hair can maintain flight ability by reducing body heat loss at low temperatures [35], which corresponds to the efficient pollination performance of *C. gigas* in low-temperature environments in this study.

This study has two limitations: first, the specific contribution of the nanoscale structure of hair (such as terminal bifurcations and nanoscale longitudinal striations) to pollen attachment has not been directly verified by scanning electron microscopy; second, the toxic effect of *C. oleifera* pollen on *A. cerana* lacks physiological data support in this study [36]. Future research will focus on why *C. gigas* is suitable for pollinating *C. oleifera* flowers, as well as the specific aspects of its unique functional characteristics. It will also combine scanning electron microscopy and atomic force microscopy to quantify the relationship between the microstructure of the pollinator’s hair and pollen adhesion force [3,31]. Hahn et al. pointed out the potential role of moths as cryptic pollinators in low-temperature flowering plants, while Mochizuki et al. found that fungus gnat-pollinated plants have specific floral characteristics [37,38]. In addition, referring to the studies on the pollination systems of early angiosperms by Luo et al., phylogenetic analysis can be used to explore the evolutionary history of the interaction between *C. oleifera* and pollinators, especially the influence of host transfer events on the differentiation of hair characteristics [39,40]. Meanwhile, drawing on the special mechanism of “parasitoid wasps transforming herbivores into pollinators” discovered by Nunes et al. can provide a new paradigm for understanding the evolution of pollination functions [41]. Finally, this study confirms the direct link between hair morphology and pollen load. We acknowledge that pollinator body size may influence pollen-carrying capacity [7]. However, our findings reveal that the larger hornets did not carry a significantly greater pollen load than the smaller Asian honey bee (*A. cerana*) (Figure 6 and Figure 8), while the superior performance of *C. gigas* is directly attributable to the highly specialized hair on its hind legs (Figure 2 and Figure 4). This indicates that, within the system studied, the morphological specialization of hair is a more critical driving factor for pollen carriage than body size alone. Future research could incorporate body size and other multivariate traits within a broader phylogenetic framework to precisely quantify the relative contribution of each trait.

This study reveals that hornets (*V. velutina*, *V. soror*) possess morphological potential for pollen carriage comparable to that of the honey bee (*A. cerana*) at the individual level. However, assessing their ecological function requires looking beyond individual traits. Hornets are efficient predators, and substantial evidence indicates that their predatory behavior can significantly reduce the abundance and visitation frequency of native pollinators, thereby exerting a strong indirect negative impact on pollination services for plants [42,43]. Particularly in invaded regions, *V. velutina* has been shown to decrease pollinator visits and seed set for various wild plants and crops through direct predation and interference [44]. This predation pressure may threaten wild bee populations upon which *C. oleifera* relies. Consequently, hornets present a potential trade-off between “morphological efficiency” and “ecological cost” within the *C. oleifera* system. Their individual pollen-carrying capacity may be offset by population-level predation pressure, leading to a negative or highly uncertain net pollination effect [45]. Future assessments should incorporate long-term monitoring to quantify the actual impact of hornet predation on the population dynamics of key pollinators like *C. gigas*. The implication of this study is that managing pollination services for *C. oleifera* requires acknowledging the functional potential of diverse flower visitors while remaining vigilant about the risk of pollination network disruption posed by high-trophic-level predators.

This study employed principal component analysis (PCA) to further reveal, at a multivariate level, the differentiation patterns among the four pollinator species in terms of morphological traits related to pollen carriage. The first two principal components cumulatively explained 74.7% of the total variance, with PC1 primarily representing traits closely associated with hair development, such as AHL, NPH, and THL. The results indicated that *C. gigas* and *A. cerana* scored higher on PC1, which corresponds to their well-developed hair morphology and confirms their status as groups with efficient pollen-collecting structures. In contrast, the two hornet species (*V. velutina* and *V. soror*) exhibited lower scores on PC1, reflecting their relatively sparse and structurally simpler hair characteristics. This clear morpho-functional grouping statistically supports the core conclusion that “hair length and density are key traits distinguishing the pollen-carrying capacity of *C. oleifera* pollinators.” Recent studies also emphasize that phenotypic traits of pollinators, such as hair structure, are important indicators for predicting pollination function and service efficacy, and that trait combinations provide greater explanatory power than single traits [46]. Notably, although hornets demonstrate certain pollen-carrying potential at the individual morphological level, their ecological role as higher-order predators may alter pollinator community structure, thereby generating complex net effects at the ecosystem level [43,45]. Therefore, the PCA results not only reinforce the morphology-based functional assessment in this study but also suggest that future screening of dominant pollinators should integrate morphological traits with species-level community interactions for a more comprehensive evaluation.

Microscopic observation of leg hair morphology provides a direct structural explanation for the interspecific differences revealed by quantitative measurements. The highly plumose, branched hairs on the legs of *C. gigas* form a three-dimensional structure that significantly increases the surface area. The inter-branch spaces may mechanically interlock with the spinose exine of *C. oleifera* pollen, thereby markedly enhancing adhesion efficiency [47]. In contrast, the sparse, linear, and simple setae on the legs of hornets are morphologically oriented more towards sensory or grooming functions rather than specialized for carrying large pollen loads. This morphological differentiation clearly reflects the distinct pollination niches of the different species: the highly specialized leg morphology of *C. gigas* represents a direct morphological adaptation to its role as an obligate, efficient pollinator of *C. oleifera*; whereas the simpler structure of hornets corresponds to their ecological role as facultative flower visitors [4]. This finding has direct implications for trait-based pollinator management. In screening for dominant pollinators, the uniquely plumose, branched hairs on the legs of *C. gigas* can serve as a key morphological identifier for rapid recognition. Future research could employ scanning electron microscopy (SEM) to further elucidate the influence of nanoscale hair structures (such as microridges or waxy layers) on pollen adhesion and investigate the matching relationship between hair morphology and the physical properties of pollen, thereby deepening the understanding of the multi-scale links between morphology and pollination function [48].

## 5. Conclusions

This study demonstrates that *C*. *gigas* is the most efficient pollinator of *C*. *oleifera* among the four wild species examined, owing to its significantly longer and denser body hairs, particularly on the legs. Hair length and density were identified as key morphological traits positively correlated with pollen load. While hornets (*V*. *velutina* and *V. soror*) possess individual-level pollen-carrying potential, their ecological role as predators may offset their pollination contribution at the community level. These findings provide a morphological basis for screening dominant pollinators and highlight the importance of integrating functional traits with ecological interactions in pollination management.

## Figures and Tables

**Table 1:** Correlation analysis between hair density, hair length, and pollen load.

Group	Pollen Load	Hair Length	Hair Density
Pollen load	1	0.545 **	0.391 **
Hair length	0.545 **	1	0.387 **
Hair density	0.391 **	0.387 **	1

Note: Asterisks denote significance levels: (**) *p* < 0.01.

## Data Availability

The original contributions presented in this study are included in the article. Further inquiries can be directed to the corresponding author.

## References

[1] Ollerton J., Winfree R., Tarrant S. (2011). How Many Flowering Plants Are Pollinated by Animals?. Oikos.

[2] Violle C., Navas M., Vile D., Kazakou E., Fortunel C., Hummel I., Garnier E. (2007). Let the Concept of Trait Be Functional!. Oikos.

[3] Roquer-Beni L., Rodrigo A., Arnan X., Klein A., Fornoff F., Boreux V., Bosch J. (2020). A Novel Method to Measure Hairiness in Bees and Other Insect Pollinators. Ecol. Evol..

[4] Gagic V., Bartomeus I., Jonsson T., Taylor A., Winqvist C., Fischer C., Slade E.M., Steffan-Dewenter I., Emmerson M., Potts S.G. (2015). Functional Identity and Diversity of Animals Predict Ecosystem Functioning Better than Species-Based Indices. Proc. R. Soc. B.

[5] Borges R.C., Padovani K., Imperatriz-Fonseca V.L., Giannini T.C. (2020). A Dataset of Multi-Functional Ecological Traits of Brazilian Bees. Sci. Data.

[6] Thorp R.W. (2000). The Collection of Pollen by Bees. Plant Syst. Evol..

[7] Woodcock B.A., Garratt M.P.D., Powney G.D., Shaw R.F., Osborne J.L., Soroka J., Lindström S.A.M., Stanley D., Ouvrard P., Edwards M.E. (2019). Meta-Analysis Reveals That Pollinator Functional Diversity and Abundance Enhance Crop Pollination and Yield. Nat. Commun..

[8] Haider M., Dorn S., Sedivy C., Müller A. (2014). Phylogeny and Floral Hosts of a Predominantly Pollen Generalist Group of Mason Bees (Megachilidae: Osmiini): Phylogeny and Floral Hosts of *Osmia*. Biol. J. Linn. Soc. Lond..

[9] Phillips B.B., Williams A., Osborne J.L., Shaw R.F. (2018). Shared Traits Make Flies and Bees Effective Pollinators of Oilseed Rape (*Brassica napus* L.). Basic Appl. Ecol..

[10] Cullen N., Xia J., Wei N., Kaczorowski R., Arceo-Gómez G., O’Neill E., Hayes R., Ashman T.-L. (2021). Diversity and Composition of Pollen Loads Carried by Pollinators Are Primarily Driven by Insect Traits, Not Floral Community Characteristics. Oecologia.

[11] Goulnik J., Plantureux S., Van Reeth C., Baude M., Mesbahi G., Michelot-Antalik A. (2020). Facial Area and Hairiness of Pollinators Visiting Semi-natural Grassland Wild Plants Predict Their Facial Pollen Load. Ecol. Entomol..

[12] Stavert J.R., Liñán-Cembrano G., Beggs J.R., Howlett B.G., Pattemore D.E., Bartomeus I. (2016). Hairiness: The Missing Link between Pollinators and Pollination. PeerJ.

[13] Smith C., Weinman L., Gibbs J., Winfree R. (2019). Specialist Foragers in Forest Bee Communities Are Small, Social or Emerge Early. J. Anim. Ecol..

[14] Switzer C.M., Russell A.L., Papaj D.R., Combes S.A., Hopkins R. (2019). Sonicating Bees Demonstrate Flexible Pollen Extraction without Instrumental Learning. Curr. Zool..

[15] Russo L., Danforth B. (2017). Pollen Preferences among the Bee Species Visiting Apple (*Malus pumila*) in New York. Apidologie.

[16] Roswell M., Dushoff J., Winfree R. (2019). Male and Female Bees Show Large Differences in Floral Preference. PLoS ONE.

[17] Ne’eman G., Shavit O., Shaltiel L., Shmida A. (2006). Foraging by Male and Female Solitary Bees with Implications for Pollination. J. Insect Behav..

[18] Wang M., Zhang Y., Li Y., Ding X., Li Y., Cai J. (2016). Preliminary Report on Superior Clones Trial of Camellia Oleifera in Guangdong Province. J. Non-Timber For. Res..

[19] Tang F., Shen D., Liu Y., Zong D., Wu Y., Teng Y. (2013). Analysis of Main Chemical Components in Oil-Tea Camellia Seed Oil and Olive Oil. J. Chin. Cereals Oils Assoc..

[20] Qiu J., Luo Y., Xu J., Wang J., Xu L. (2013). Industrial Development Strategy and Technical Paths for Camellia Oleifera in Guizhou. Guizhou For. Sci. Technol..

[21] Li X., Chen Y., Li B., Zhang S., Chen S. (2019). Preliminary Study on Improved Variety Selection of Camellia Oleifera in Hilly Areas of Eastern Guangdong. For. Reconnaiss. Des..

[22] Deng Y., Yu X., Luo Y. (2010). Effect of Pollinating Insects on Fruit and Seed Setting of Camellia Oleifera in Central-South China. Acta Ecol. Sin..

[23] Deng Y., Yu X., Lei R., Huang J., Xu Y., Yang W., Xiang J. (2009). Pollination Biological Characteristics of Camellia Oleifera. J. Non-Timber For. Res..

[24] He X., Cai S., Xiong Y., Han G., Chen Y., Huang L., Wu Q. (2010). Main Pollinating Insect Species and Flower-Visiting Behavior in Camellia Oleifera Forests in Fujian Province. Fujian For. Sci. Technol..

[25] Luo J., Zhao C., Huang H., Jiang X. (2014). Investigation on Diversity of Pollinating Insects for Camellia Oleifera in Guangxi. Guangxi For. Sci..

[26] Ollerton J. (2017). Pollinator Diversity: Distribution, Ecological Function, and Conservation. Annu. Rev. Ecol. Evol. Syst..

[27] Rader R., Cunningham S.A., Howlett B.G., Inouye D.W. (2020). Non-Bee Insects as Visitors and Pollinators of Crops: Biology, Ecology, and Management. Annu. Rev. Entomol..

[28] Li H., Orr M.C., Luo A., Dou F., Kou R., Hu F., Zhu C., Huang D. (2021). Relationships between Wild Bee Abundance and Fruit Set of *Camellia oleifera* Abel. J. Appl. Entomol..

[29] Huang D., He B., Gu P., Su T., Zhu Z. (2017). Discussion on Current Situation and Research Direction of Pollination Insects of Camellia Oleifera. J. Environ. Entomol..

[30] Wei W., Li X., Wei X., Lu W., Yang X., Zheng X. (2017). Review of Species, Nesting and Pollination Behaviors of Pollinating Insects in *Camellia* spp.. Guangxi For. Sci..

[31] Jia X., Zhou F., Pan J., Zhao Y., Zhang W., Wang K., Shu J. (2025). Morphology and Distribution Characteristics of Hind Leg Setae in Five Species of Bee Pollinators of *Camellia oleifera*. Chin. J. Ecol..

[32] Mehmet S. (2011). Pollen Quality, Quantity and Fruit Set of Some Self-Compatible and Self-Incompatible Cherry Cultivars with Artificial Pollination. Afr. J. Biotechnol..

[33] Zhuang R. (1988). China’s Oil-Tea Camellia.

[34] Qiu J. (2016). Study on Pollinating Insects of Camellia Plants in Southwest China. Ph.D. Thesis.

[35] Peters M., Peisker J., Steffan-Dewenter I., Hoiss B. (2016). Morphological Traits Are Linked to the Cold Performance and Distribution of Bees along Elevational Gradients. J. Biogeogr..

[36] Li Z. (2023). Analysis of the Mechanism of Poisoning in Bees Collecting *Camellia oleifera* Nectar and Pollen. Ph.D. Thesis.

[37] Mochizuki K., Kawakita A. (2018). Pollination by Fungus Gnats and Associated Floral Characteristics in Five Families of the Japanese Flora. Ann. Bot..

[38] Hahn M., Brühl C.A. (2016). The Secret Pollinators: An Overview of Moth Pollination with a Focus on Europe and North America. Arthropod-Plant Interact..

[39] Luo S.-X., Zhang L.-J., Yuan S., Ma Z.-H., Zhang D.-X., Renner S.S. (2018). The Largest Early-Diverging Angiosperm Family Is Mostly Pollinated by Ovipositing Insects and so Are Most Surviving Lineages of Early Angiosperms. Proc. R. Soc. B.

[40] Luo S.-X., Yao G., Wang Z., Zhang D., Hembry D.H. (2017). A Novel, Enigmatic Basal Leafflower Moth Lineage Pollinating a Derived Leafflower Host Illustrates the Dynamics of Host Shifts, Partner Replacement, and Apparent Coadaptation in Intimate Mutualisms. Am. Nat..

[41] Nunes C.E.P., Maruyama P.K., Azevedo-Silva M., Sazima M. (2018). Parasitoids Turn Herbivores into Mutualists in a Nursery System Involving Active Pollination. Curr. Biol..

[42] Rojas-Nossa S., Calviño-Cancela M. (2020). The Invasive Hornet Vespa Velutina Affects Pollination of a Wild Plant through Changes in Abundance and Behaviour of Floral Visitors. Biol. Invasions.

[43] Rojas-Nossa S.V., O’Shea-Wheller T.A., Poidatz J., Mato S., Osborne J., Garrido J. (2023). Predator and Pollinator? An Invasive Hornet Alters the Pollination Dynamics of a Native Plant. Basic Appl. Ecol..

[44] Monceau K., Maher N., Bonnard O., Thiery D. (2013). Predation Pressure Dynamics Study of the Recently Introduced Honeybee Killer Vespa Velutina: Learning from the Enemy. Apidologie.

[45] Heineke M.R., Kimbro D.L., Zabin C.J., Grosholz E.D. (2023). Harnessing Trophic Cascades to Improve Foundation Species Restoration: A Meta-analysis. Ecosphere.

[46] Bartomeus I., Cariveau D.P., Harrison T., Winfree R. (2018). On the Inconsistency of Pollinator Species Traits for Predicting Either Response to Land-use Change or Functional Contribution. Oikos.

[47] Koch L., Lunau K., Wester P. (2017). To Be on the Safe Site—Ungroomed Spots on the Bee’s Body and Their Importance for Pollination. PLoS ONE.

[48] Amador G.J., Matherne M., Waller D., Mathews M., Gorb S.N., Hu D.L. (2017). Honey Bee Hairs and Pollenkitt Are Essential for Pollen Capture and Removal. Bioinspir. Biomim..

